# A retrospective study demonstrating the growth patterns and the pseudoprogression temporal classification after stereotactic radiosurgery for sporadic vestibular schwannomas

**DOI:** 10.1038/s41598-025-03095-4

**Published:** 2025-05-25

**Authors:** Amr M N El-Shehaby, Wael A Reda, Khaled M Abdel Karim, Ahmed M Nabeel, Reem M Emad Eldin, Ahmed H Alazzazi, Sameh R Tawadros

**Affiliations:** 1 Gamma Knife Center Cairo, Nasser Institute Hospital, Cairo, Egypt; 2 Neurosurgery Department, Faculty of Medicine, Ain Shams University, Cairo, Egypt; 3 Clinical Oncology Department, Faculty of Medicine, Ain Shams University, Cairo, Egypt; 4 Radiation Oncology Department, National Cancer Institute, Cairo University, Cairo, Egypt; 5 Neurosurgery Department, Faculty of Medicine, Benha University, Qalubya, Egypt; 6 Extended Modular Program, Ain Shams University, Cairo, Egypt

**Keywords:** Vestibular Schwannoma, Stereotactic radiosurgery, Sporadic, Pseudoprogression, Growth, Transient expansion, Radiotherapy, Neurosurgery

## Abstract

Background and objectives Vestibular schwannomas are known to demonstrate tumor expansion, commonly referred to as pseudoprogression, after SRS. It is critical to differentiate between true tumor progression and pseudoprogression as this may entail performing an unnecessary intervention, such as surgery or repeat radiosurgery. This study aims to identify the fate of tumor enlargement that may occur after SRS for vestibular schwannomas and to propose a management algorithm for vestibular schwannoma enlargement after SRS. Methods In this retrospective study, we included 171 patients with sporadic vestibular schwannomas who showed tumor enlargement after SRS. The mean dose was 11.9 Gy (10–12 Gy). The mean tumor volume was 4.1 cc (0.1–19.7 cc). More than half of the tumors were Koos grade 4 (Koos 1: 8 (5%), 2: 20 (12%), 3: 43 (25%), 4: 100 (58%)). Volumetric changes and clinical outcomes were recorded. Different progression patterns were recorded according to the tumor volume changes (TVC) and the timings of TVC. Results The pseudoprogression rate among the patients who showed tumor enlargement after SRS was 83% (142/171). The mean follow-up duration was 64 months (12–241 months). The actuarial progression-free survival at 5-,7- and 10-years was 95%, 92%, and 90%, respectively. The mean follow-up duration was 64 months (12–241 months). The mean TVC at progression (TVCp) was 72% (11–439%). The mean time to tumor progression was 13 months (2–160 months) and the mean duration of TVC was 15 months (2–164 months). Late pseudoprogression (after 3 years) occurred in 30 patients (21%). In early PP, there was a shorter duration of volume change, and a presence of CLC. In true progression, there was a bigger TVCp, a bigger TVCf and a bigger tumor volume at the final follow-up (TVf). Clinical decline was observed with tumor enlargement in 36% of the patients, but in most of them, improvement occurred without the need for tumor intervention. Conclusion GKR for VS is associated with radiation-induced tumor enlargement in a group of patients. Pseudoprogression may occur beyond 5 years after treatment. A more conservative approach may be adopted in most vestibular schwannomas that exhibit tumor enlargement after SRS, as in most cases, they will eventually be controlled.

**Supplementary Information:**

The online version contains supplementary material available at 10.1038/s41598-025-03095-4.

## Introduction

Vestibular schwannomas are estimated to comprise 8% of intracranial tumors^1^. More than 90% are of the sporadic type^2^. Depending on the radiological and clinical context, management may be observation, surgery or SRS. Tumor control after SRS ranges from 90 to 99% depending on but not limited to the tumor size, follow-up duration, and dose^3^. Vestibular schwannomas are known to demonstrate transient expansion after SRS, known as pseudoprogression^4–20^. Pseudoprogression is defined as a temporary increase in tumor size followed by tumor shrinkage or stability. It is critical to differentiate between true tumor progression and pseudoprogression, as this may entail performing an unnecessary intervention, such as surgery or repeat radiosurgery.

In this study, we aim to demonstrate the fate of tumor enlargement after SRS for sporadic vestibular schwannomas and associated clinical manifestations and suggest a management algorithm.

## Methods

This was a retrospective study. Between June 2001 and June 2022, 1667 patients with sporadic VS were treated by single-session GK. From these, 1411 patients were available for follow-up. Tumor progression was defined as a volume increase of > 10% from the initial tumor volume. Inclusion criteria were patients with sporadic vestibular schwannomas, showing more than 10% increase in tumor volume after SRS and with more than one year follow-up after recorded tumor progression. Exclusion criteria were patients with NF2 and less than one year follow-up. One hundred and seventy-one patients fulfilled the inclusion criteria in this study. The patient demographics and treatment parameters are summarized in Tables 1 and 2, respectively.

**Table 1:** Patient demographics.

Age (mean)	47 ± 12 (20–75)
Sex Males Females	67 (39%) 104 (61%)
Symptoms	No. of patients (%)
Accidental	5 (3)
Tinnitus	102 (60)
Hearing deficit	152 (89)
Facial pain	4 (2)
Facial numbness	36 (21)
Facial weakness	18 (11)
Facial tics	3 (2)
Vertigo	48 (28)
Disequilibrium	46 (27)
Bulbar	1 (1)
Hydrocephalus	10 (6)
Shunt before GK	7 (4)
Previous surgery	24 (14)
*Koos grade* Grade 1 Grade 2 Grade 3 Grade 4	8 (5%) 20 (12%) 43 (25%) 100 (58%)

**Table 2:** Treatment parameters.

Parameter	Mean (range)
Tumor volume (cc)	4.1 ± 4.9(0.1–19.7)
Prescription dose (Gy)	11.9 ± 0.4 (10–12)
Cover (%)	98 ± 2.2 (90–100)
Prescription isodose (%)	51 ± 3.9 (50–78)
PIV (cc)	4.8 ± 4.6 (0.1–19.2)
PTV (cc)	5.8 ± 5.3 (0.2–22.9)
Mean dose (Gy)	16 ± 1.1 (13.3–19.2)
Max. dose (Gy)	23 ± 1.5 (15.4–24.8)
Integral dose (mJ)	75.7 ± 69.2 (1.9–286.8)
SI	0.8 ± 0.1 (0.3–0.96)
GI	3.5 ± 0.7 (2.3–6.1)

True progression, synonymous with treatment failure, was considered if there was a progressive increase in tumor size and/or progressive neurological deterioration, requiring some form of tumor intervention. Pseudoprogression was defined as tumor progression after treatment followed by tumor stability or shrinkage without the need for tumor intervention. All methods were carried out in accordance with appropriate guidelines. Approval was obtained from the relevant IRB (Ethical Committee of the Specialized Medical Centers (SMC)). Informed consent was obtained from all subjects and/or their legal guardian(s). This study was conducted and reported in accordance with the STROBE guidelines for observational studies. The STROBE checklist was used to ensure comprehensive and transparent reporting of study design, data collection, and analysis.

### Follow-up volumetric analysis

Volumetric assessment was performed. A thin slice contrasted MRI was done then imported and co-registered in Leksell GammaPlan software (version 11.3.2) with MRI done on the day of treatment. The tumor was then drawn on all slices and the volume were determined from dose-volume histogram, the same way as the treatment planning day. From the follow-up images, the tumor volumes (TV), tumor volume changes from the original volume (TVC) and the timings of TVC were recorded. Other image changes were reported, including edema (T2 hyperintense signal) and central loss of contrast (CLC).

### Statistical analysis

Tumor volume at the time of treatment (TVt), tumor volume (peak volume) at progression (TVp), tumor volume change at progression (TVCp), tumor volume at the final follow-up (TVf), and tumor volume change at the final follow-up (TVCf) were reported. The time to progression was also reported. Serial volume measurements were made after treatment. Mean values were tested using independent t-test and ANOVA. Categorical variables were tested for by X^2^ or Fisher exact test. Secondary outcomes such as edema, hydrocephalus, cyst formation, and clinical outcome were also tested for. Case matching for tumor volume was done. Statistical analysis was done using SPSS version 26 (SPSS Inc, Chicago, Illinois, USA).

## Results

The mean follow-up duration was 64 months (12–241 months). The mean TVCp was 72% (11–439%). The mean time to tumor progression was 13 months (2–160 months) and the mean duration of TVC was 15 months (2–164 months).

### Progression patterns

Progression patterns were identified according to tumor behavior: Type 1 in which tumor enlargement was followed by eventual shrinkage (95/171;56%), Type 2 in which tumor enlargement was followed by stability (47/171;27%) and Type 3 in which there was tumor enlargement with a progressive increase in tumor size on serial images (29/171;17%). In a univariate analysis of the progression pattern type we found the following significant factors: in Type 1: CLC with progression 85/95 (90%) (*p* < 0.0001), smaller TVCp (45.7% vs. 69% vs. 161%) (*p* < 0.0001), earlier progression (17 months vs. 28 months vs. 56 months) (*p* < 0.0001), shorter duration of volume change (19 months vs. 39 months) (*p* < 0.0001). In Type 3: Cyst formation 4/7 (57%) (p 0.01) and a shorter follow-up (*p* < 0.0001). In a multivariate analysis only, a shorter follow-up was significant (p 0.02) for Type 3.

There was also a biphasic progression pattern in which the tumor showed two progression peaks with a period of tumor stability or regression in between then eventual regression or stability. This pattern was identified in 6 patients. The first peak occurred at a mean of 12 months (7–34 months) and the second peak at 47 months (24–76 months). The mean TVt was 5.5 cc (0.7–14.3 cc). In univariate analysis, follow-up duration (141 months vs. 79 months) (p 0.04) was significantly associated with this phenomenon. The tumor eventually shrank in 4 patients and remained stable in 2 patients.

### Tumor enlargement

Pseudoprogression was identified in 142 patients (83%). A temporal classification of tumor pseudoprogression was devised categorizing them into 3 groups:

*Early*: tumor enlargement occurred during the first year after treatment (Supplementary Fig. 1). It was observed in 85 patients (60%) starting at a mean of 7 months (2–12 months).

*Intermediate*: tumor enlargement occurred after the first year and within 3 years of treatment (Supplementary Fig. 2). It was observed in 27 patients (19%) starting at a mean of 22 months (13–36 months).

**Fig. 1:**
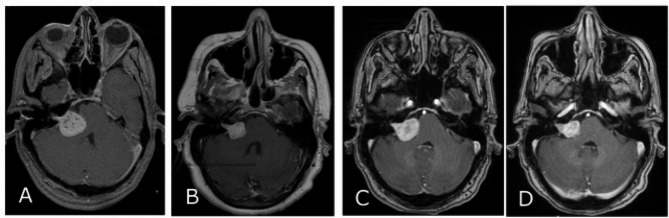
Late PP **A** At the time of treatment; 7.4 cc tumor was treated with 11 Gy to the 50% isodose with 99% cover, **B** 30 months after treatment; 4.5 cc tumor volume (−39% TVC), **C** 40 months after treatment; 5.5 cc tumor volume (−25% TVC), **D** 79 months after treatment; 2.7 cc tumor volume (−64% TVCf).

*Late*: tumor enlargement occurred after 3 years of treatment (Fig. 1). It was found in 30 patients (21%) starting at a mean of 59 months (37–162 months).

True progression was found in 29 patients (17%) at a median of 56 months (19–152 months). In 23 patients, serial radiological tumor progression was established. In 6 patients progressive neurological deterioration, without further radiological progression, necessitated surgical intervention for the tumor.

A univariate analysis of the progression type was done. In early PP, there was a shorter duration of volume change, type 1 pattern and a presence of CLC. In true progression, there was a bigger TVCp, a bigger TVt and a bigger TVCf (Supplementary Tables 1 and 2).

### Hydrocephalus

Hydrocephalus occurred after GK in 18 patients (11%) predominantly in intermediate pseudoprogression (p 0.04). Fourteen patients were found to have pseudoprogression and 4 were found to be true progression. In 14 patients VP shunt was enough to correct the patient’s clinical deterioration with no need for further intervention. Four patients required additional tumor debulking or excision.

### Cyst formation

Cyst formation was observed in 7 patients (4%). One patient underwent surgical tumor intervention because of clinical deterioration and radiological tumor and cyst progression, followed by GK treatment for the residual tumor. In two patients, spontaneous cyst regression occurred, while one patient underwent cyst aspiration, and no further intervention was required as the tumor itself eventually shrank. In three patients, GK re-treatment was performed. Cyst formation was predominantly associated with late PP (p 0.02).

### Tumor control

**Fig. 2:**
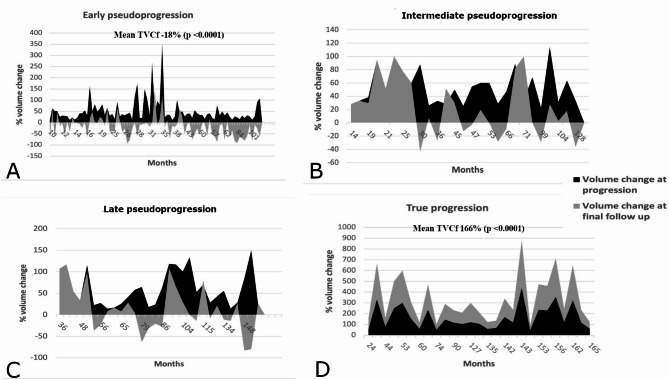
**A** Early PP; TVCf was − 18% (−93–145%) (p <0.0001),  **B** Intermediate PP; TVCf was 26% (−43–114%), **C** Late PP; TVCf was 14% (−83–117%), **D** True progression; TVCf was 166% (47–439%) (p <0.0001).

**Fig. 3:**
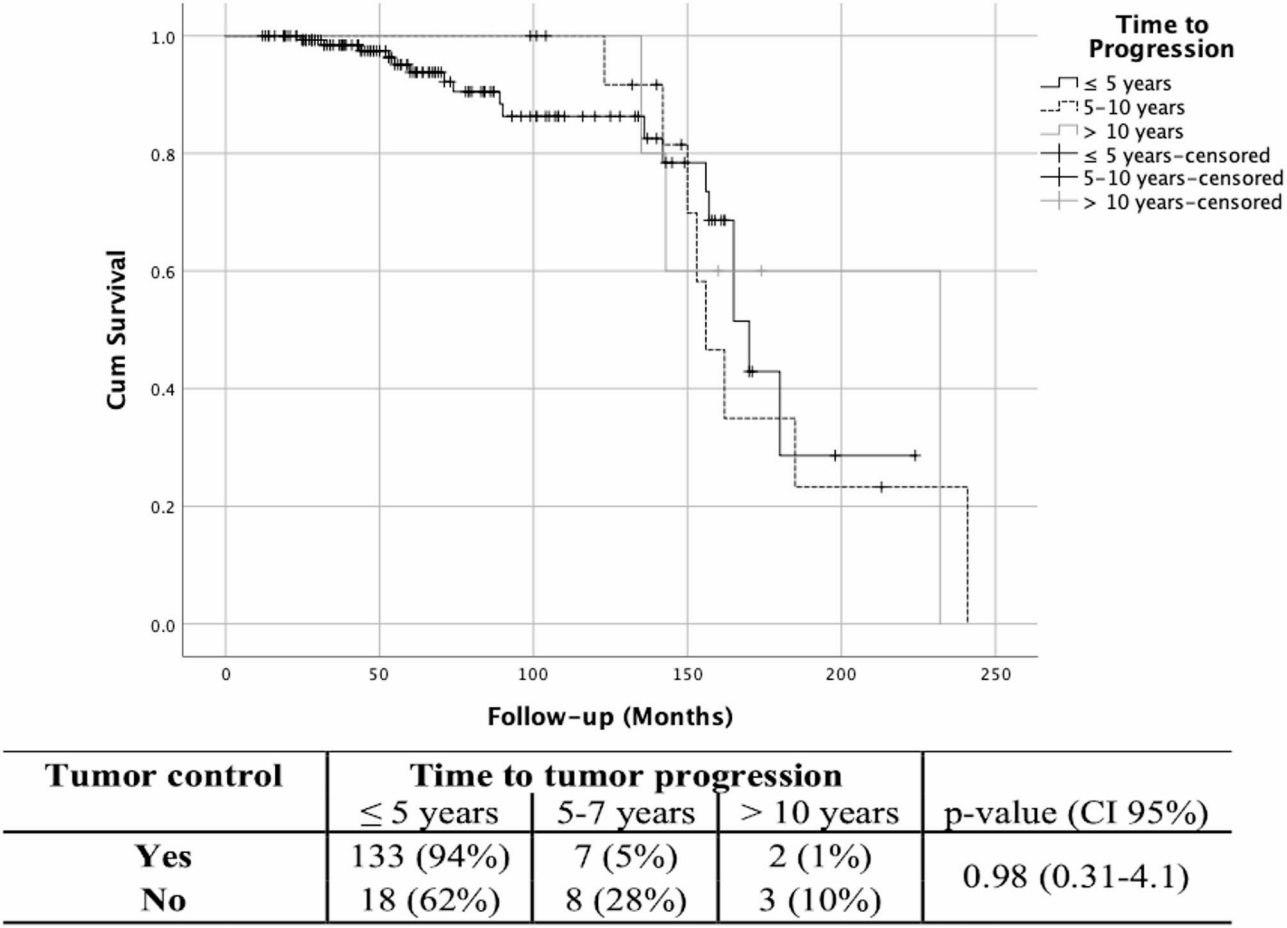
Kaplan-Meier analysis for treatment failure in vestibular schwannomas after gamma knife radiosurgery. The table shows the timing of treatment failures at different follow-up intervals.

The pseudoprogression rate among the patients who showed tumor enlargement after SRS was 83% (142/171). The actuarial progression-free survival at 5-,7- and 10-years was 95%, 92%, and 90%, respectively. Most cases of treatment failure were recorded within 5 years from GK (Fig. 2). The mean time to true progression was 56 months (Supplementary Table 1). In a univariate analysis, factors favoring tumor control were CLC with progression 112/142 (79%) (*p* < 0.0001), shorter follow up (72 vs. 126 months) (< 0.0001), lesser TVCp (53% vs. 161%) (< 0.0001), lesser TVf 4.4 cc vs. 8.5 cc (*p* < 0.0001), TVCf − 3.2% vs. 165.9% (*p* < 0.0001), and earlier progression 21 months vs. 56 months (*p* < 0.0001). In a multivariate analysis, CLC with progression (p 0.02), follow-up duration (p 0.03) and, TVCf (*p* < 0.0001) were significant (Table 3). The highest tumor volume reduction was found in patients with early PP (*p* < 0.0001) while the most volume increase was seen in patients with true tumor progression (*p* < 0.0001) (Fig. 3).

**Table 3:** Analysis of factors affecting tumor control.

Parameters	Univariate analysis	Multivariate analysis	
	p-value (95% CI)	p-value (95% CI)	Effect size
Age	0.48 (0.99–1.02)	-	-
Sex	0.68 (0.77–1.51)	-	-
Koos grade	0.55 (0.6–1.32)	-	-
CLC with progression	***< 0.0001*** (1.97–4.47)	***0.02*** (0.02–21)	0.112
Tumor volume	0.32 (0.96–1.14)	-	-
Prescription dose	0.4 (0.13–2.26)	-	-
TVp	0.37 (0.99–1.04)	-	-
TVCp	***< 0.0001*** (0.99–1)	0.88 (0.01–0.02)	0.006
TVf	0.62 (0.94–1)	-	-
TVCf	***< 0.0001*** (0.99–1)	***< 0.0001*** (0.002–0.003)	0.003
Time to progression	***< 0.0001*** (0.97–0.98)	0.46 (−0.001-0.002)	0.001
Follow-up duration	***< 0.0001*** (0.98–0.99)	***0.03*** (0.001–0.002)	0.001

ROC analyses were done to determine the cut-off values for true tumor progression and clinical progression/decline. The cut-off for TVCf for true progression was 51% (< 0.0001), and the AUCwas 0.96. Fifty patients (29%) showed more than 50% volume increase at the final follow-up, but only 8 patients had large tumors with a volume of more than 10 cc (p 0.8). The cut-off for TVCf associated with clinical decline was 40% (< 0.0001), and the AUC was 0.88. The cut-off for TVCp associated with clinical decline was 61% (p 0.66) but was not significant. (Supplementary Fig. 3).

### Clinical status

New or worsened clinical status at the time of tumor enlargement was observed in 61 patients (36%) (Table 4). Neurological deterioration was due to hydrocephalus in 18 patients (30%), due to cyst formation in 3 patients (5%), and in the remaining patients due to tumor compression. In 41/61 patients (67%), neurological improvement occurred, including 14 patients after VP shunt, two patients with cysts that experienced spontaneous regression, and one patient who underwent cyst evacuation. The remaining patients improved spontaneously or with the aid of steroid therapy. In a univariate analysis, the factors determining the final clinical decline were cyst formation (p 0.03), greater TVCp (*p* < 0.0001), greater TVf (p 0.01), greater TVCf (*p* < 0.0001), radiological progression (*p* < 0.0001), and longer time to progression (*p* < 0.0001). In a multivariate analysis, TVf (*p* < 0.0001) and radiological progression (*p* < 0.0001) were significant (Tables 5).

**Table 4:** New or worsened manifestations at time of tumor enlargement.

Symptoms	No. of patients (%)
Facial pain	3 (2)
Facial numbness	12 (7)
Facial tics	5 (3)
Vertigo	18 (11)
Disequilibrium	39 (23)
Bulbar	2 (1)
Hydrocephalus	18 (11)

**Table 5:** Analysis of factors affecting clinical outcome.

Parameters	Univariate analysis	Multivariate analysis	
	p-value (95% CI)	p-value (95% CI)	Effect size
Cyst	0.03 (0.09–0.91)	0.47 (−0.1–0.21)	0.056
TVCp	< 0.0001 (0.99–1)	0.43 (0–0.0001)	0
TVf	0.01 (0.92–0.99)	***< 0.0001*** (0.006–0.02)	0.012
TVCf	< 0.0001 (0.99–1)	0.48 (−0.001-0)	0
Radiological progression	< 0.0001 (0.002–1)	***< 0.0001*** (−1.01–0.79)	−0.92
Time to progression	< 0.0001 (0.97–0.98)	0.35 (−0.002–0.001)	−0.001

### Salvage treatment after GK failure

**Fig. 4:**
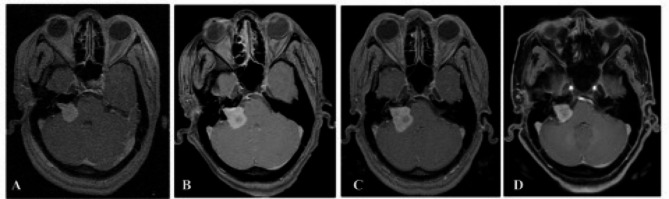
GK salvage treatment **A** At the time of treatment; 3.3 cc tumor was treated with 12 Gy to the 50% isodose with 97% cover, **B** 6 months after treatment 4.8 cc tumor volume (44% TVCp), **C** 61 months after treatment; 7.2 cc tumor volume (118% TVCf). GK re-treatment was performed to a 7.2 cc tumor that was treated with 12 Gy to the 50% isodose with 97% cover, **D** 70 months after 2nd GK treatment; 4.6 cc tumor volume (−36% TVC).

Twenty-seven patients underwent salvage treatment. Nineteen patients (71%) underwent GK re-treatment alone, six patients (22%) underwent surgery alone, and two patients (7%) had surgery then GK re-treatment. The median follow-up duration after salvage treatment was 68 months (4–160 months). The tumor volume according to salvage treatment was; mean 5.4 cc (0.5–15.8 cc) for GK re-treatment alone (Fig. 4), mean 14.9 cc (5–30 cc) for surgery alone, and mean 12.6 cc (12–13.2 cc) for surgery followed by GK re-treatment. The five patients who underwent surgery alone were lost to follow-up. Tumor control was achieved in 19/21 patients (90%). The actuarial tumor control was 94% at 5 years. The tumors shrank in 15 patients (71%), remained stable in 4 patients (19%), and progressed in 2 patients (10%). The two patients with tumor progression are still being followed up.

## Discussion

### Tumor control

The definition of tumor control after SRS was traditionally regarded as either stability or shrinkage of tumor size on follow-up images. However, this does not apply to all tumor types, as some tumors may transiently swell after SRS. This is in addition to the lack of universally accepted criteria for tumor control after SRS; most depend on an absolute or relative change in tumor size. To avert this, it has been proposed that tumor control be defined as the absence of the need for a second tumor intervention^3,21,22^. Considering the cohort of patients with sporadic VS treated by GK, from which the cases of this study were taken, the tumor control would be 98% (1382/1411), including the patients that turned out to be pseudoprogression. The tumor control of VS treated by SRS is commonly reported to exceed 90%^3,21^. We observed that tumors showing CLC were less likely to exhibit true progression. Earlier studies reported similar findings^5^. Tumors that exhibited earlier tumor enlargement, usually within the first two years, were more likely to be controlled, as we found earlier progression was significantly associated with tumor control. True progression was significantly associated with greater volume changes in this study, specially more than 100%. Matsuo et al. found that a greater than two-fold increase in tumor size was associated with continued tumor growth.

Most cases of treatment failure in the current study were recorded within 5 years from GK. However, most were earlier patients in our practice, in whom treatment failure may have been determined prematurely. The current study found that the mean time to treatment failure was determined much later, around 5 years from treatment. Later on we advocated more careful assessment of the clinical status of the patients and weighing conservative management against surgical intervention for the tumor, in addition to resorting to non-tumor-based interventions for other complications such as cysts or hydrocephalus. Most reports determined that treatment failure occurs at around 5 years^6,13,23–26^.

### Pseudoprogression

Pseudoprogression is defined as tumor expansion following SRS followed by either tumor stability or regression^14^. The reported incidence of vestibular schwannoma enlargement after SRS is 3–80%^5–7,9–20,27^ (Table 6). In the current series, VS enlargement was present in 12% of treated cases. The high variability may be because of the criteria and the method of measurement used to assess tumor enlargement. Some might rely on computer-generated volume measurements or manually obtained through two-dimensional measurements on MRI images and then using a formula. They then report a percentage change from the original size, which can vary from 10 to 25%. Others would report an increase in size of a few millimeters in one or more dimensions using only two-dimensional measurements^3,28^. Volume measurements appear to be more accurate, as reported by several studies^13,15,29^, which we used in the current study. The current study used the 10% volume increase as a threshold indicating tumor enlargement. Snell et al., using computational geometrical methods, indicated that volume changes of less than 10% are within the error levels with volumetric measurements^30^. Further analysis of our cohort showed that 91% (155 patients) had a volume increase of more than 20%, and only 9% (16 patients) had a volume increase of less than 20%. This shows little difference between the 10% and 20% volume change thresholds. The rationale for using a lower threshold was being more meticulous, which is in the patient’s interest, so although almost 90% of the tumors surpassed the 20% threshold, it also means that 10% would not have been detected as growth, giving the patients a false sense of security. It also means that, with the lower threshold, if the patient continues to follow up and turns out to show continuous tumor progression, then the true progression can be detected earlier than with a higher threshold, and intervention will be done in a timely manner.

**Table 6:** Studies reporting on pseudoprogression.

Study	No. of patients	No. of patients with tumor progression	Tumor size measurement	Tumor volume (cc)	Criterion for significant tumor growth	Incidence of PP	TVCp	Time to progression (months)	Duration of VC	Treatment failure determination
Pollock et al. 2006^20^	208	30 (14%)	Linear	1.5 median	≥ 2 mm mean diameter increase	24 (12%)	75% median (26–280%)	NR	NR	Progressive tumor enlargement on serial imaging
Hasegawa et al. 2006^8^	254	42 (17%)	Linear	5.6 mean (0.2–36.7)	≥ 2 mm mean diameter increase	25 (10%)	NR	9 median (3–21) 18 median (9–51) 12 median (2–69)	NR	Any neurological deterioration or cyst formation
Nagano et al. 2008^15^	100	74 (74%)	Volumetric	2.7 mean (0.1–13.2)	≥ 10% volume increase	NR	47% mean (0–613%)^#^	6.4 mean	NR	NR
Hayhurst el al 2012^19^	75	26 (35%)	Volumetric	1.7 median (0.3–8.2)	> 10% volume increase	17 (23%)	PP: 23% median (10–81%) Persistent growth: 67% (10–507%)	PP: 6 mean (6–9) GKF: 3, 36 and 48	NR	Symptomatic tumor growth
Mindermann et al. 2014^17^	235	21 (9%)	Volumetric	1.9	≥ 20% volume increase	4 (2%)	NR	41	NR	≥ 20% volume increase 3–4 years after treatment
Matsuo et al. 2015^7^	44	29 (66%)	Volumetric	2.4 (0.4–9)	≥ 20% volume increase	24 (55%)	NR	NR	NR	Increased volumes of more than two-fold and continued growth for at least 2 years
Breshears el al 2019^11^	118	74 (63%)	Volumetric	0.74 median (0.34–1.77)	Significant volume increase at any time following treatment	52 (44%)	49% mean	13 mean	90% resolved at 83 months	Significant, monotonic volume increase over time following treatment
Wage et al. 2021^6^	112	42 (38%)	Calculated volume	1.4 mean (failure) 1.5 mean (control)	> 10% volume increase	33 (29%)	*Type 1:134% mean (119–166%) Type 2:156% mean (144–180%)	Type 1: 5.5 median (4.8–6) Type 2: 31 median (27.5–36) GKF: 57 median (29–123)	Type 1: 17 median (15–22.3) Type 2: 55 median (51–59.5)	Continued tumor growth and progressive symptoms at least 2 years after treatment or after growth without progressive symptoms of at least 5 years
Rueß et al. 2023^32^	63	18 (29%)	Calculated volume	1.5 median (0.1–8.6)	> 20% volume increase	18 (29%)	57% mean	18 median (4–61) 36 median (20–61)	12.5 median (5–82)	> 40% volume increase with additional continuous growth beyond 48 months
Present study	1411	171 (12%)	Volumetric	4.1 mean (0.1–19.7)	> 10% volume increase	142 (10%)	72% mean (11–439%)	13 mean (2–160)	15 mean (2–164)	Progressive neurological deterioration (excluding hydrocephalus or cyst) or serial tumor progression with clinical deterioration

Conventional tumor response criteria, such as Macdonald and RECIST, were originally developed for oncological purposes, mainly for malignant and systemic tumors. However, these criteria have limited value in the response assessment of benign tumors, as these are slow-responding tumors. Consequently, volume change occurs after extended periods and in a portion of patients only. More recently, modified RANO criteria were developed for meningiomas^31^ and vestibular schwannomas^32^. They have proposed that the cut-off for progressive disease be a volume increase of more than 40%. This value aligns with our findings, which suggest a cut-off for true progression and clinical progression/decline of 51% and 40%, respectively. It should be noted that pseudoprogression is less common in meningiomas, reportedly in 5–11%^33^, so the suggested cut-off values for vestibular schwannomas should be more generous. Our results also show that most tumors with these volume changes were less than 10 cc volume, probably because larger tumors may have required intervention.

Several hypotheses have been proposed for the development of pseudoprogression. It has been suggested that radiation-induced inflammation results in increased vascular permeability^34^. There is the associated release of cytokines, VEGF, and other mediators, as well as tumor infiltration by macrophages^35,36^. Other explanations include the induction of an immune response by radiation resulting in tumor infiltration by immune cells and disruption of the blood-brain barrier^37^.

Previously it was thought pseudoprogression occurred as early as 2 months and as late as 36 months after SRS^14,17,38^. Early pseudoprogression is commonly reported^8,19,39^. 50% of the patients in the current study had early pseudoprogression, and the mean time for pseudoprogression was 13 months. Wage et al. reported that there were two types of pseudoprogression, one as early as 6 months after treatment and one later at around 3 years from treatment^14^. Among the identified progression patterns, Type 1 had a shorter duration of pseudoprogression, and Type 2 lasted longer. In the current study, we identified a third type of pseudoprogression (late), which occurred later than 3 years, at around 5 years from treatment. The early type had a significantly shorter duration of swelling (15 months) compared to the other two types (39 and 44 months, respectively). Recently, Balossier et al.^40^ reported on radiological outcome using clustering to determine the different response patterns to gamma knife treatment of vestibular schwannomas. They attempted to predict the response trajectories in the five patterns they identified. In their study, pattern 2 showed either stability or continuous progression and was the most likely to show treatment failure. The remaining patterns were more likely destined for tumor control. Central loss of contrast is a common postradiosurgical finding in VS, which usually occurs in the first year and is followed by a return of central contrast enhancement^41–43^. We found that most tumors showed CLC in the early type PP and progression pattern type 1. The early type of PP had lesser volume change compared to other types at progression and final follow-up. In addition, the early type was all without any clinical consequences. Type2 progression was mentioned in two previous studies, occurring in 19% and 29% of patients with pseudoprogression^13,44^. In the current study, it was found in 27% of patients. A more complex progression pattern, referred to in this study as biphasic, in which the tumor showed two instances of progression along the timeline of their follow-up, separated by periods of tumor stability or even shrinkage. Similarly, Matsuo et al. mentioned 3 patients with a bimodal peak at one and three years^15^in their study. This phenomenon suggests that if the tumor progresses even after initial tumor regression, one should not rush to consider this treatment failure.

### Complications

Post-SRS hydrocephalus has been reported in 1–19% of cases^45–50^. In the current study, it was observed in 11% of the patients. However, this cohort is a subgroup from the total number of treated patients, meaning the incidence is likely much lower. We found hydrocephalus was associated with larger tumors. Lee et al. found that post-SRS hydrocephalus occurs within 3–4 years at around the time of tumor expansion^51^. We found hydrocephalus occurred predominantly in late PP at around 2 years after treatment.

The incidence of cyst formation after SRS may be as low as 2%. Pikis et al. reported cyst formation in 6% of patients^17^. We observed this phenomenon in 4% of patients. Two patients did not undergo any intervention and regressed spontaneously, and one patient only required cyst evacuation.

### Salvage treatment

Repeat SRS for VS after treatment failure has been reported with a tumor control rate of 85–95%. Better control rates were observed after the second radiosurgical treatment^52–56^. This study found a similar control rate to the first treatment. It may be related to the longer follow-up duration compared to previous studies. As with primary radiosurgery treatments, treatment failures tend to occur after many years. Slightly higher rates of facial and trigeminal neuropathy were reported.

Surgery after radiosurgical treatment has been a matter of controversy and debate for years. Unfortunately, we could not record the difficulty or ease of the surgeries after treatment failure because they were not performed at our center, as we are a stand-alone gamma knife center. It was suggested that better results may be associated with subtotal or near-total as opposed to gross total resection^13^.

### Management algorithm

**Fig. 5:**
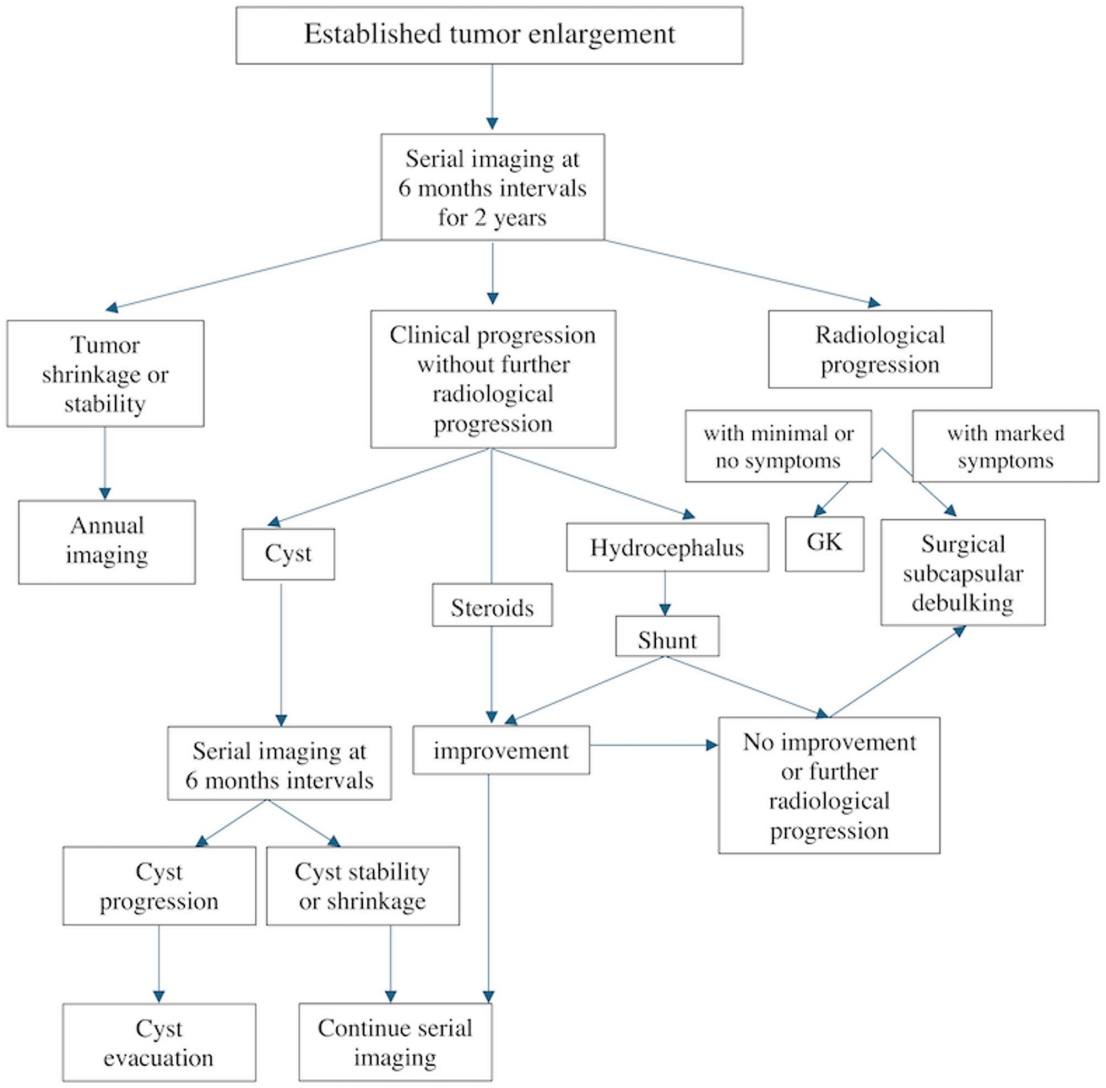
Management algorithm.

We observed that two-thirds of the patients in whom clinical deterioration occurred at the time of tumor progression were effectively managed expectantly or with a VP shunt. It is also possible that a more patient wait-and-see strategy may have been adopted in some of these patients, especially for the smaller-sized tumors and asymptomatic patients (Fig. 5). More recent studies have adopted more stringent criteria for treatment failure and a more patient approach to tumor enlargement after SRS (Table 6). In the current study, we have demonstrated that pseudoprogression can occur beyond 5 years and as late as 10 years after treatment. We suggest that guided by the patient’s clinical condition, we proceed with only serial imaging if the symptoms are mild or absent. In the case of gross clinical deterioration, the least invasive strategy is adopted, as in the case of shunt insertion for hydrocephalus or cyst evacuation. In the presence of radiological progression on serial MRIs with minimal or no symptoms, depending on the size of the tumor we may adopt a wait-and-see strategy for smaller tumors or proceed for tumor intervention for larger tumors. The suggested cut-off values for tumor volume change should not be taken at face value as more tolerance should be practiced in smaller, asymptomatic, or minimally symptomatic tumors. Finally, the timing of PP may be an important guide in management. It was noted that early PP should not be of concern as most cases will regress, while intermediate and late PP will either follow a regressive or stable path. Tumors that showed true progression exhibited continuous serial radiological progression on follow-up, unlike those with PP, which remained stable over a period of at least one year.

### Limitations

The retrospective nature of the study, related to selection bias and lost follow-up, may affect its strength. A reasonably long follow-up duration, as well as a record of the patients that experienced treatment failure and subsequent salvage treatment, with also an acceptable follow-up, may make up for this.

It may be argued that some patients that remained stable after initial enlargement can grow in the future. However, the results suggest they are more likely to shrink in the long run, specially in intermediate and late PP.

## Conclusion

GKR for VS is associated with radiation-induced tumor enlargement in a group of patients. A more conservative approach may be adopted in most vestibular schwannomas that exhibit tumor enlargement after SRS, as they will eventually be controlled in most cases. Pseudoprogression may occur after 5 years.

## Electronic supplementary material

Below is the link to the electronic supplementary material.

Supplementary Material 1

## Data Availability

Data will be made available upon reasonable request. An Excel spreadsheet with all deidentified data and software code on which the conclusions of the paper rely will be available for inspection and verification during the peer-review process. Further inquiries can be directed to the corresponding author.

## Abbreviations

GK: Gamma knife
CLC: Central loss of contrast
PP: Pseudoprogression
TTE: Transient tumor expansion
GI: Gradient index
RANO: Response Assessment in Neuro-Oncology
RECIST: Response Evaluation Criteria for Solid Tumors
ROC: Receiver Operating Characteristic
SI: Selectivity index
SRS: Stereotactic radiosurgery
TV: Tumor volume
TVC: Tumor volume change
TVCp: Tumor volume change at progression
TVCf: Tumor volume change at the final follow-up
TVf: Tumor volume at the final follow-up
TVp: Tumor volume at progression
TVt: Tumor volume at the time of treatment
VEGF: Vascular endothelial growth factor
