# Supplementary material for: A retrospective study demonstrating the growth patterns and the pseudoprogression temporal classification after stereotactic radiosurgery for sporadic vestibular schwannomas
Source: Sci Rep. 2025 May 25;15:18187. doi: 10.1038/s41598-025-03095-4 (PMC12104479; doi:10.1038/s41598-025-03095-4)
Supplement: Supplementary file 1 — Supplementary Material 1 [file 41598_2025_3095_MOESM1_ESM.docx]

Supplementary Table 1. Tumor enlargement sequelae and relationship to post-treatment radiological and clinical changes

|  | **Early pseudo-progression** | **Intermediate pseudo-progression** | **Late pseudo-progression** | **True progression** | **p value** |
| --- | --- | --- | --- | --- | --- |
| **TVCp (%)** | 51.2±52.5 (11-350) | 52±25.8 (22-114) | 60.6±40.9 (14-150) | 160±107.7 (47-439) | <0.0001 |
| **Time to progression (months)** | 6.9±1.6 (3-12) | 22.1±8.7 (13-36) | 58.8±30.6 (36-160) | 56.3±36.5 (19-152) | <0.0001 |
| **TVp (cc)** | 7.7±6.5 (0.3-25.5) | 6.5±5.8 (0.2-19.8) | 6.1±7.3 (0.7-30) | 8.3±7.7 (0.4-25) | 0.52 |
| **Duration of pseudo-progression (months)** | 15.2±20.1 (5-164) | 39.6±37.4 (5-157) | 43.5±29.8 (6-111) |  | <0.0001 |
| **TVCf (%)** | -18±45.9 (-93-145) | 23.2±42.5 (-43-114) | 14.3±53.2 (-83-117) | 165.9±104.9 (47-439) | <0.0001 |
| **TVf (cc)** | 4±4.3 (0.1-22.6) | 5.2±5.3 (0.2-19.1) | 4.8±6.6 (0.3-30) | 8.5±8.3 (0.1-31.6) | 0.005 |
| **CLC at tumor growth** | 82/85 (97%) | 17/27 (63%) | 13/30 (43%) | 7/29 (24%) | <0.0001 |
| **Edema** | 22/85 (26%) | 7/27 (26%) | 4/30 (13%) | 7/29 (24%) | 0.55 |
| **Hydrocephalus after GK** | 6/85 (7%) | 7/27 (26%) | 2/30 (7%) | 3/29 (10%) | 0.04 |
| **Cyst formation** | 1/85 (1%) | 0/27 (0) | 2/30 (7%) | 4/29 (14%) | 0.02 |
| **Progression pattern**  **Type 1**  **Type 2**  **Type 3** | 67 (79%)  18 (21%)  0 (0) | 12 (44%)  15 (56%)  0 (0) | 16 (53%)  14 (47%)  0 (0) | 0 (0)  0 (0)  29 (100%) | <0.0001 |
| **Clinical status at time of progression** | 50 stable (59%)-35 worse (41%) | 17 stable (63%)-10 worse (37%) | 26 stable (87%)-4 worse (13%) | 17 stable (59%)-12 worse (41%) | 0.04 |
| **Final clinical status** | 81 stable-5 died (unrelated causes) | 25 stable-1 worse | 27 stable-3 worse | 1 stable-28 worse | <0.0001 |
| **Follow up duration (months)** | 58±44 (12-224) | 70±39 (14-128) | 81±43 (36-174) | 126±56 (24-241) | <0.0001 |

(mean values and ranges are presented)

Supplementary Table 2. Treatment parameters' relationship to tumor enlargement

|  | **Early pseudo-progression** | **Intermediate pseudo-progression** | **Late pseudo-progression** | **True progression** | **p value** |
| --- | --- | --- | --- | --- | --- |
| **Prescription dose** | 11.8±0.4 (11-12) | 11.9±0.4 (11-12) | 11.9±0.5 (10-12) | 11.9±0.3 (11-12) | 0.47 |
| **Tumor volume at treatment** | 5.6±4.9 (0.1-19.7) | 4.3±3.5 (0.1-11.1) | 4.4±4.8 (0.3-19.7) | 4.1±4.9 (0.1-16.5) | 0.34 |
| **Isodose** | 51.3±4 (50-70) | 51.5±4.3 (50-70) | 51±5.1 (50-78) | 50.1±0.9 (50-55) | 0.57 |
| **Cover** | 98.1±1.9 (91-100) | 98.7±1.2 (96-100) | 97.2±2.5 (91-100) | 95.7±2.3 (90-100) | <0.0001 |
| **PIV** | 5.6±4.9 (0.1-19.2) | 4.2±3.5 (0.1-10.9) | 4.2±4.7 (0.3-18.7) | 3.9±4.5 (0.1-15.1) | 0.21 |
| **PTV** | 6.6±5.6 (0.2-22.9) | 4.9±3.8 (0.3-12.3) | 5.1±5.6 (0.4-22.9) | 4.8±5.3 (0.2-18.2) | 0.25 |
| **Mean dose** | 15.8±1.1 (13.6-18.2) | 16.3±1.1 (14.5-19.2) | 16±1 (13.3-17.9) | 16±0.9 (14.1-18.3) | 0.26 |
| **Maximum dose** | 23.1±1.5 (17.2-24.8) | 23.2±1.7 (17.1-24.2) | 23.4±1.8 (15.4-24.8) | 23.8±0.7 (22-24.4) | 0.19 |
| **Integral dose** | 87±75 (1.9-286.8) | 65.4±52.9 (2.1-162.8) | 67±69.2 (5.4-278.6) | 62.1±65.5 (1.9-286.8) | 0.23 |
| **Selectivity index** | 0.8±0.1 (0.4-1) | 0.8±0.1 (0.4-0.9) | 0.8±0.1 (0.5-0.9) | 0.7±0.2 (0.3-0.9) | 0.12 |
| **Gradient index** | 3.7±0.8 (2.3-5.9) | 3.7±0.8 (2.6-6.1) | 3.3±0.5 (2.4-4.2) | 3.3±0.5 (2.6-4.3) | 0.008 |
| **Koos grade**  **1**  **2**  **3**  **4** | 2 (2%)  10 (12%)  18 (21%)  55 (65%) | 3 (11%)  2 (7%)  8 (30%)  14 (52%) | 1 (3%)  4 (13%)  9 (30%)  16 (53%) | 2 (7%)  4 (14%)  8 (30%)  15 (52%) | 0.68 |

(mean values and ranges are presented)


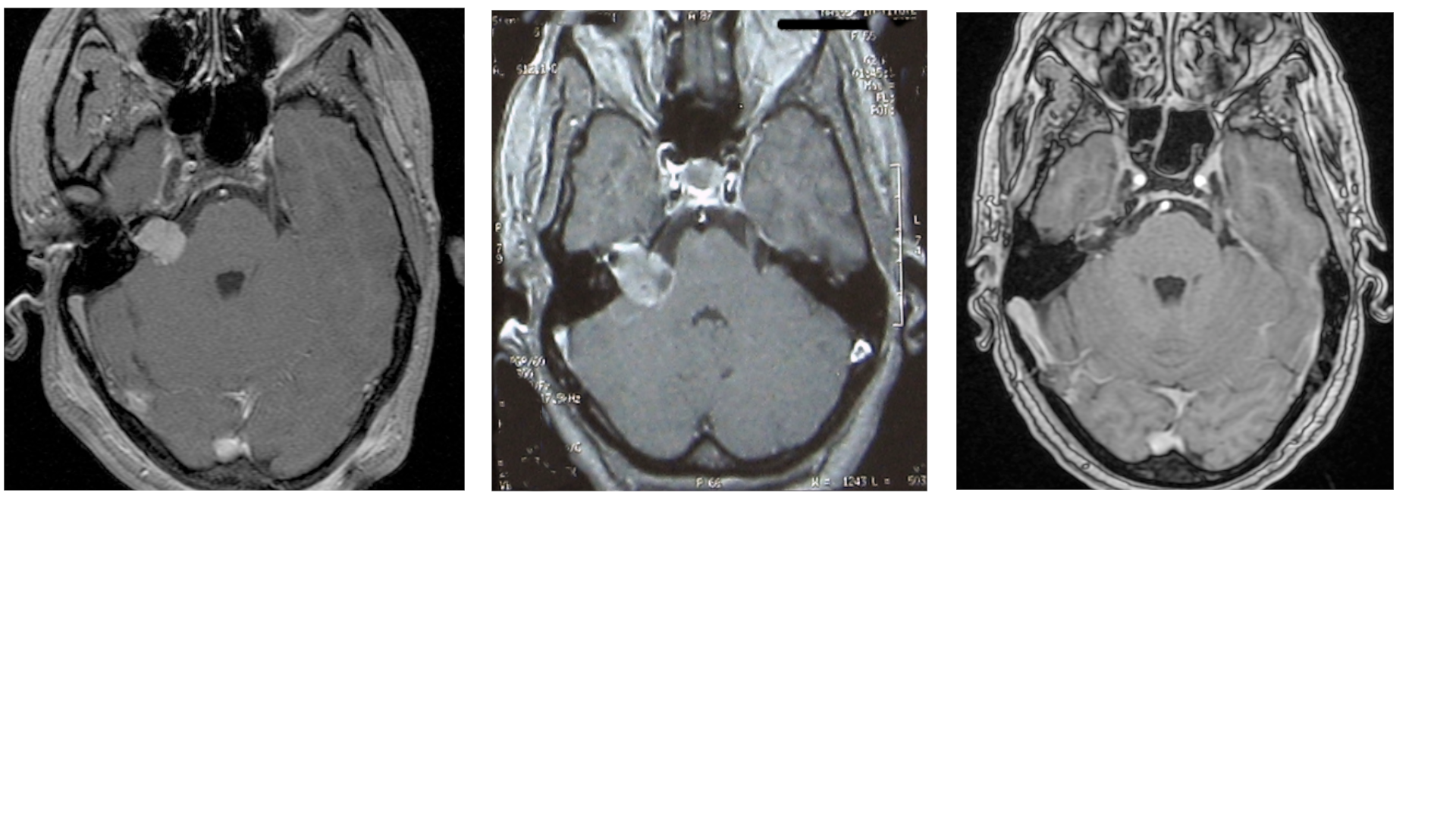


Supplementary Figure 1. **Early PP** (A) At the time of treatment; 2.4 cc tumor was treated with 12 Gy to the 50% isodose with 93% cover, (B) 6 months after treatment; 5 cc tumor volume (108% TVCp), (C) 224 months after treatment, 0.9 cc tumor volume (-63% TVCf).


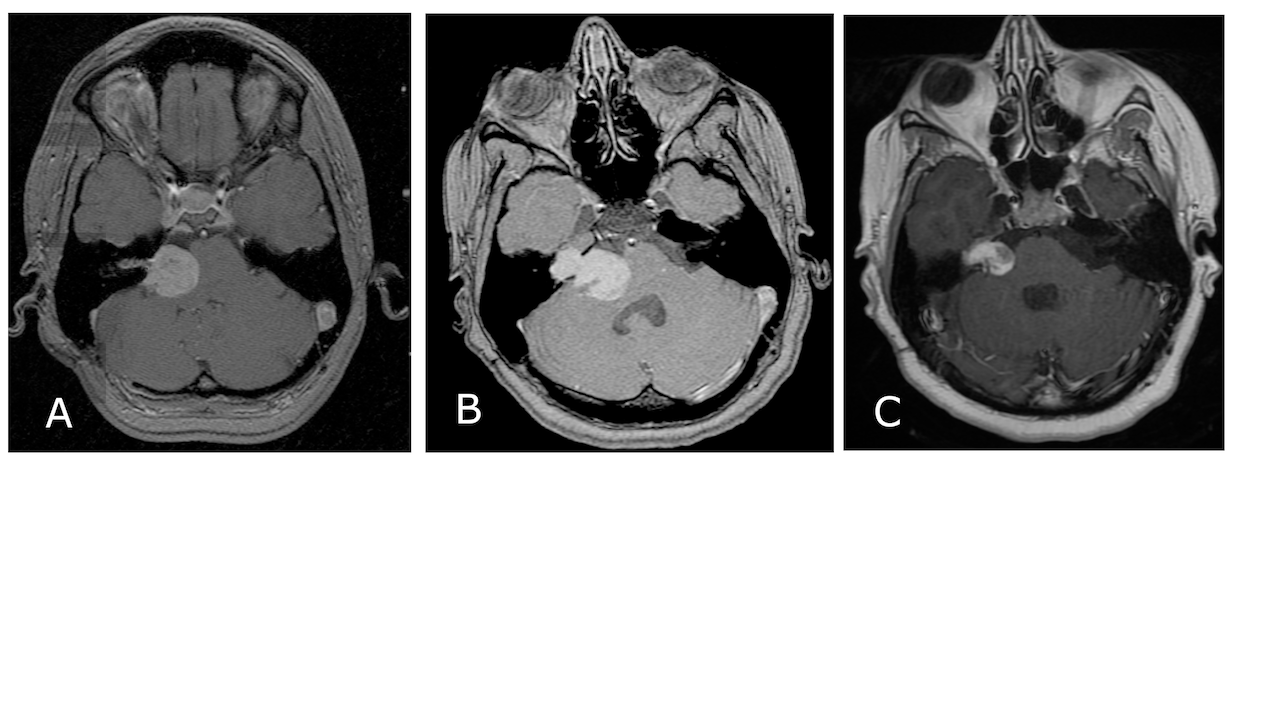


Supplementary Figure 2. **Intermediate PP** (A) At the time of treatment; 6.9 cc tumor was treated with 12 Gy to the 50% isodose with 97% cover, (B) 34 months after treatment; 9.3 cc tumor volume (33% TVCp), (C) 128 months after treatment; 4.4 cc tumor volume (-36% TVCf).


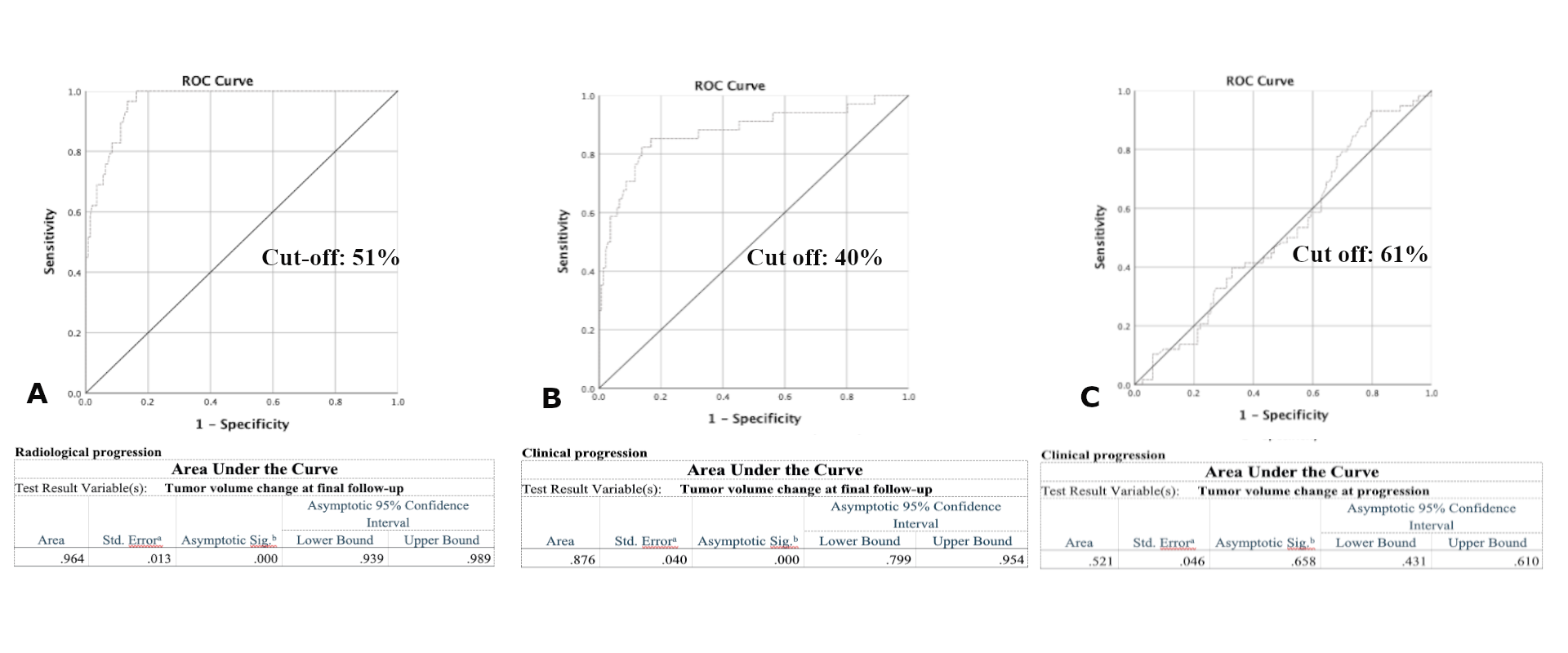


Supplementary Figure 3. ROC analyses with cut-off values for radiological (true) progression and clinical progression/decline associated with tumor volume and volume change at the time of maximum tumor progression and at the final follow-up. **(A)** The cut-off for TVCf for true radiological progression was 51% (<0.0001). **(B)** The cut-off for TVCf associated with clinical decline was 40% (<0.0001). **(C)** The cut-off for TVCp associated with clinical decline were 61% (p 0.66).
